# Construction of luciferase-expressing *Neospora caninum* and drug screening

**DOI:** 10.1186/s13071-024-06195-8

**Published:** 2024-03-08

**Authors:** Fei Wang, Yangfei Xue, Yanqun Pei, Meng Yin, Zhepeng Sun, Zihui Zhou, Jing Liu, Qun Liu

**Affiliations:** 1 National Animal Protozoa Laboratory, College of Veterinary Medicine, China Agricultural University, Beijing, 100193 China; 2 Key Laboratory of Animal Epidemiology of the Ministry of Agriculture, College of Veterinary Medicine, China Agricultural University, Beijing, 100193 China

**Keywords:** *Neospora caninum*, Firefly luciferase, CRISPR/Cas9, TAK-632, Inhibition

## Abstract

**Background:**

*Neospora caninum* is an apicomplexan parasite that is particularly responsible for abortions in cattle and neuromuscular disease in dogs. Due to the limited effectiveness of currently available drugs, there is an urgent need for new therapeutic approaches to control neosporosis. Luciferase-based assays are potentially powerful tools in the search for antiprotozoal compounds, permitting the development of faster and more automated assays. The aim of this study was to construct a luciferase-expressing *N. caninum* and evaluate anti-*N. caninum* drugs.

**Methods:**

Luciferase-expressing *N. caninum* (Nc1-Luc) was constructed using clustered regularly interspaced short palindromic repeats (CRISPR)-associated protein 9 (CRISPR/Cas9). After testing the luciferase expression and phenotype of the Nc1-Luc strains, the drug sensitivity of Nc1-Luc strains was determined by treating them with known positive or negative drugs and calculating the half-maximal inhibitory concentration (IC_50_). The selective pan-rapidly accelerated fibrosarcoma (pan-RAF) inhibitor TAK-632 was then evaluated for anti-*N. caninum* effects using Nc1-Luc by luciferase activity reduction assay and other in vitro and in vivo studies.

**Results:**

The phenotypes and drug sensitivity of Nc1-Luc strains were consistent with those of the parental strains Nc1, and Nc1-Luc strains can be used to determine the IC_50_ for anti-*N. caninum* drugs. Using the Nc1-Luc strains, TAK-632 showed promising activity against *N. caninum*, with an IC_50_ of 0.6131 μM and a selectivity index (SI) of 62.53. In vitro studies demonstrated that TAK-632 inhibited the invasion, proliferation, and division of *N. caninum* tachyzoites. In vivo studies showed that TAK-632 attenuated the virulence of *N. caninum* in mice and significantly reduced the parasite burden in the brain.

**Conclusions:**

In conclusion, a luciferase-expressing *N. caninum* strain was successfully constructed, which provides an effective tool for drug screening and related research on *N. caninum*. In addition, TAK-632 was found to inhibit the growth of *N. caninum*, which could be considered as a candidate lead compound for new therapeutics for neosporosis.

**Graphical Abstract:**

**Figure:**
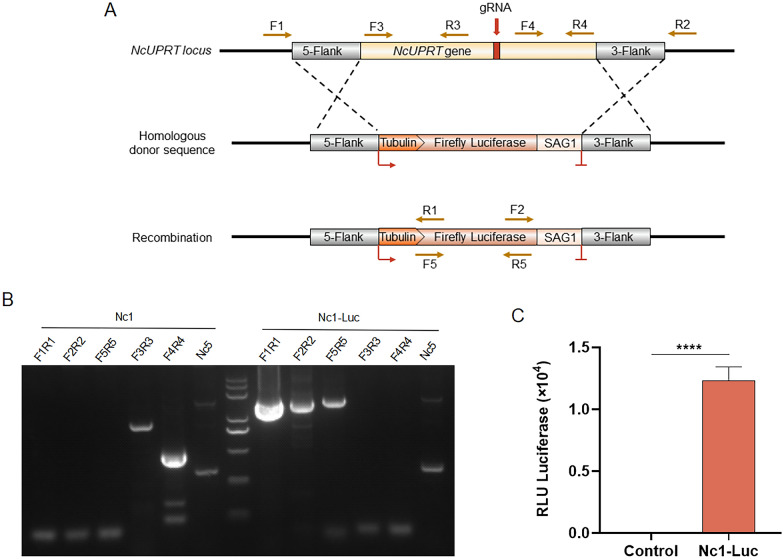

**Supplementary Information:**

The online version contains supplementary material available at 10.1186/s13071-024-06195-8.

## Background

*Neospora caninum* is an obligate intracellular protozoan parasite belonging to the phylum Apicomplexa, which is known to cause abortions in cattle and neuromuscular diseases in dogs, resulting in significant economic losses [1, 2]. Currently, there are no specific drugs to combat neosporosis, and existing treatments are unsatisfactory due to their low efficiency [3]. Therefore, novel therapies are urgently needed to reduce abortions caused by *N. caninum* and vertical transmission of the parasite.

To identify novel therapeutics against *N. caninum*, high-throughput screening of a library of candidate compounds is required to determine their efficacy in inhibiting the growth of the parasite. To date, several methods have been used to assess the growth of *N. caninum* and to screen for effective anti-*N. caninum* compounds, including classical microscopic parasite counting using Giemsa staining or immunofluorescence assays (IFA) [4, 5], parasite DNA detection using real-time polymerase chain reaction (PCR) [6], and radioactive 3H-uracil incorporation-based quantification [7]. Each of these methods has unique advantages, but certain limitations have restricted their application. For example, parasite counting and real-time PCR are labor-intensive, time-consuming, and unsuitable for screening large numbers of drugs [8]. They do not reflect parasite viability, but only parasite numbers [9]. Furthermore, radioactive isotopes have potential health hazards and safety concerns [10].

Reporter genes such as green fluorescent protein (GFP), β-galactosidase (β-gal), and luciferase (Luc) have facilitated the discovery of drugs targeting protozoan parasites. Among the different reporter genes, luciferase is especially attractive due to its high efficiency, absence of background activity in host cells, and bioimaging ability. Recombinant protozoan parasites that express luciferase have been used to screen drugs against a wide range of protozoa, including *Toxoplasma*, *Plasmodium*, *Leishmania*, and *Trypanosoma* [11]. Due to the use of automated plate readers, these recombinant protozoan parasites can make the screening of antiprotozoal compounds more suitable for standardized and high-throughput purposes [12]. They can also be used to visualize the progression of parasitic infections in vivo. Saeij et al. [13] examined the in vivo growth, dissemination, and reactivation of strains of the protozoan parasite *Toxoplasma gondii* using recombinant parasites that stably express luciferase.

In recent years, small-molecule inhibitors targeting intracellular protein kinases have gained prominence due to their low molecular weight, good membrane permeability, and high specificity. A broad range of small-molecule inhibitors and targets are currently being investigated in protozoan parasites [14]. TAK-632, a selective pan-rapidly accelerated fibrosarcoma (pan-RAF) inhibitor, was initially developed for the treatment of melanoma [15, 16]. Subsequent studies found that it could target the receptor-interacting protein kinases RIPK1 and RIPK3 against necroptosis [17, 18]. However, it is unclear whether TAK-632 exerts anti-*N. caninum* effects, and the mechanism underlying the clearance of intracellular parasites is unknown.

Here, a recombinant *N. caninum* strain expressing firefly luciferase (Nc1-Luc) was constructed and evaluated for infectivity in vitro and in vivo. The study demonstrated that Nc1-Luc has promising potential for use in studies of high-throughput drug screening assays. In addition, TAK-632 was evaluated for anti-*N. caninum* effects using Nc1-Luc by luciferase activity reduction assay and other in vitro and in vivo studies. The results showed that TAK-632 could effectively inhibit the growth of *N. caninum* and is a promising drug candidate for the treatment of neosporosis.

## Methods

### Parasites, host cells, and compounds

African green monkey kidney (Vero) cells and human foreskin fibroblasts (HFF) were obtained from the Cell Bank of the Chinese Academy of Sciences (Shanghai, China). The cells were cultured in Dulbecco’s modified Eagle medium (DMEM, M&C Gene Technology Ltd., China) supplemented with 8% fetal bovine serum (FBS, Thermo Fisher Scientific, USA). *Neospora caninum* tachyzoites were maintained by continuous passage in Vero cells in DMEM supplemented with 2% FBS. Both cells and parasites were incubated at 37 °C in 5% CO_2_. To collect freshly egressed and unpolluted parasites used in the study, *N. caninum* tachyzoites were cultured continuously for 3–4 days until a large number of tachyzoites were observed under the microscope, and the cells were scraped off with a cell scraper (Biologix Biotechnology Co., Ltd., China) and broken repeatedly with a 1 ml syringe (Zhiyu Medical Equipment Co., Ltd., China). The cell debris was then filtered with a 5 µm filter (Taoyuan Medical Chemical Instrument Factory, China) and centrifuged at 2800 rpm for 8 min to obtain the precipitation of parasites.

Atovaquone (20 mM, catalog number: T1491), pyrimethamine (100 mM, catalog number: T0849), penicillin G potassium (198.66 mM, catalog number: T5602), and TAK-632 (50 mM, catalog number: T1886) were purchased from Topscience (Shanghai Topscience Biotechnology Co., Ltd., Shanghai, China) and dissolved in 100% dimethyl sulfoxide (DMSO, Thermo Fisher Scientific, USA). Aliquots were stored at −80 °C until use.

### Animals and ethics approval

BALB/c mice used in animal experiments were purchased from Beijing Vital River Laboratory Animal Technology Co., Ltd. (Beijing, China, license no. SCXK [Jing] 2021-0006). All experiments on mice were performed in strict accordance with the recommendations of the Guide for the Care and Use of Laboratory Animals of the Ministry of Science and Technology of China. All procedures were approved by the Institutional Animal Care and Use Committee of China Agricultural University (under the certificate of Beijing Laboratory Animal employee ID CAU-AW31901202-2-1).

### Plasmid construction

All primers can be found in Additional file 1: Table S1. Images of the plasmid construction are shown in Additional file 2: Figure S1.

The *N. caninum* uracil phosphoribosyltransferase (NcUPRT)-targeting clustered regularly interspaced short palindromic repeats (CRISPR) plasmid pNc_Cas9CRISPR::sgNcUPRT and the plasmid pNcUPRT::GFP were constructed by Yang [19]. To construct the plasmid pEASY-Luc containing the firefly luciferase expression cassette under the *T. gondii tubulin* promoter, the firefly luciferase reporter plasmid pGL3-Basic Vector (Promega Biotech Co., Ltd., China) was used as a template for amplifying the firefly luciferase fragment using primers Luc-F/R. Additionally, the plasmid pEASY-mCherry, preserved in our laboratory, served as the template for amplifying the vector fragment containing the *Tgtubulin* promoter and *TgSAG1* terminator, employing primers T-S-F/R [19, 20]. Information on the *Tgtubulin* promoter and *TgSAG1* terminator is listed in Additional file 3: Table S2. To construct the plasmid pNcUPRT::Luc with upstream and downstream regions directly adjacent to the *NcUPRT* gene sequence, the plasmid pNcUPRT::GFP served as a template for amplifying the pUC19 vector fragment containing the upstream and downstream homology arms of the *NcUPRT* gene, employing primers GJ-F/R. Additionally, the plasmid pEASY-Luc constructed above was used as a template for amplifying the firefly luciferase expression cassette using primers T-Luc-S-U-F/R. All plasmids were constructed using the One Step Cloning Kit (Vazyme Biotech Co., Ltd., China).

### Generation of a transgenic line of *N. caninum* expressing a firefly luciferase

The CRISPR-associated protein 9 (CRISPR/Cas9) system was used to generate *N. caninum* expressing a firefly luciferase. The plasmid pNc_Cas9CRISPR::sgNcUPRT containing NcUPRT-target guide RNA (gRNA) (sequence: 5′-GCGGGCGAGTCGATGGAAAG-3′) was used for gene disruption. Plasmid pNcUPRT::Luc with a luciferase expression cassette was used for homologous recombination. Nc1 strains were transfected with plasmid pNc_Cas9CRISPR::sgNcUPRT and the linearized product of plasmid pNcUPRT::Luc by electroporation. The linearized product was amplified using the primers Luc-Linearize-F/R listed in Additional file 1: Table S1 and the plasmid pNcUPRT::Luc as a template. Specifically, the linearized product of the pNcUPRT::Luc plasmid and the pNc_Cas9CRISPR::sgNcUPRT plasmid were sterilized by heating in a metal bath at 65 °C for 30 min. Nc1 tachyzoites were washed with Cytomix buffer (prepared in our laboratory) and centrifuged at 2800 rpm for 8 min. The supernatant was discarded. The Nc1 tachyzoites, together with the linearized product of plasmid pNcUPRT::Luc and plasmid pNc_Cas9CRISPR::sgNcUPRT, were then transferred to electroporation cuvettes (4 mm, Bio-Rad, USA), and Cytomix buffer was added to the electroporation cuvettes in a constant volume of 750 µl. The mixture ratio of plasmid pNcUPRT::Luc and plasmid pNc_Cas9CRISPR::sgNcUPRT was 5:1. Electroporation was performed using an electroporator (Gene Pulser Xcell, Bio-Rad, USA) under the following conditions: 2050 V voltage, 25 F capacitance, and ∞ resistance. After transfection, the parasites were grown in a fluorodeoxyribose (FUDR, Sigma-Aldrich, USA) selective medium. Independent clones were selected when a large number of parasites were observed under a microscope. Specificity for firefly luciferase cassette integration and *NcUPRT* deletion was identified by PCR. All primers used for identification are listed in Additional file 1: Table S1. Primers F1/R1 and F2/R2 were designed to verify homologous integration upstream and downstream of the *NcUPRT* gene locus. Primer F5/R5 was designed to verify the integration of firefly luciferase cassettes. Primers F3/R3 and F4/R4 confirmed the deletion of the *NcUPRT* gene locus. The *Nc5* gene was used as a *N. caninum*-specific gene.

### Luciferase activity assay

HFF cells were seeded in 96-well plates and cultured at 37 °C in 5% CO_2_. After the cells reached 100% confluence, each well was inoculated with 2 × 10^4^ Nc1-Luc strain and cultured at 37 °C in 5% CO_2_ for 72 h. Then the medium in the wells was removed. According to the Firefly Luciferase Reporter Gene Assay Kit (Beyotime Biotech, Shanghai, China), 100 µl of reporter gene cell lysate was added to each well and incubated at 37 °C for 30 min. Then, 100 µl of luciferase detection reagent was added to each well and protected from light for 1–2 s. The relative light units (RLUs) of the parasites were detected using a fluorescence microplate reader (Tecan, Infinite M200 PRO, Männedorf, Switzerland) with a luminescence wavelength of 560 nm. The detection interval was set to 2 s and the detection time to 10 s.

For the compound assay, parasite-infected cells in each well were treated with twofold serial dilutions of the compounds. The concentrations of the compounds were as follows: atovaquone (ranging from 20 μM to 6.1 × 10^–4^ μM, with 16 gradients), pyrimethamine (ranging from 100 μM to 0.0244 μM, with 13 gradients), penicillin G potassium (ranging from 198.66 μM to 1.552 μM, with eight gradients), and TAK-632 (ranging from 20 μM to 0.3125 μM, with six gradients). In addition, 0.1% DMSO was used as a negative control. Inhibition rate = [(RLU_DMSO_ – RLU_compound_)/RLU_DMSO_] × 100%. Means ± standard deviations (SD) of the results of three independent experiments containing three replicates for each condition are shown. The half-maximal inhibitory concentration (IC_50_) indicates the concentration of compounds that inhibited parasite growth by 50% and was calculated using the log (inhibitor) versus normalized response-variable slope in GraphPad Prism 8.0.2 software (San Diego, CA, USA).

### Immunofluorescence assays

HFF cells in 12-well plates were fixed with 4% paraformaldehyde (Sigma-Aldrich, USA) overnight at 4 °C, then permeabilized with 0.25% Triton X-100 (Sigma-Aldrich, USA) for 30 min and blocked with 3% bovine serum albumin (BSA, Sigma-Aldrich, USA) for 1 h at room temperature. Subsequently, the cells were incubated with primary antibodies. The shapes of Nc1 tachyzoites were labeled using rabbit anti-NcSRS2 (1:100, polyclonal antibody prepared and stored in our laboratory). The sequence of NcSRS2 was obtained from ToxoDB (http://toxodb.org/toxo/, accession number: NCLIV_033250) and is listed in Additional file 3: Table S2. Cells were then incubated with Cy3-conjugated goat anti-rabbit immunoglobulin G (IgG: 1:100, Sigma-Aldrich, USA). Nuclear DNA was stained with Hoechst dye (concentration: 10 µg/ml, dilution: 1:100, Sigma-Aldrich, USA). All antibodies were incubated at 37 °C for 1 h. Images were obtained using an inverted fluorescence microscope (Olympus IX73, Olympus Corp., Tokyo, Japan). The excitation wavelengths of the Cy3 and Hoechst dyes were 550 and 346 nm, respectively.

### Plaque assay

Parasites (300 tachyzoites per well) were inoculated into HFF cells in 12-well plates and grown for 9 days at 37 °C in 5% CO_2_. The medium was then removed and the cells were washed three times with phosphate-buffered saline (PBS, Biotopped Technology Co., Ltd., Beijing, China). Subsequently, cells were fixed with 4% paraformaldehyde overnight at 4 °C. The cells were then washed three times with PBS and stained with 0.2% crystal violet (Macgene Biotechnology Co., Ltd., Beijing, China) for 2 h at room temperature. The stained wells were air-dried and scanned using a Canon digital scanner (Model F917500, Japan). The plaques in each well were counted manually, and at least 10 plaques per well were randomly selected to measure the pixel size of each plaque using pixels in Photoshop CC software (Adobe, USA) to represent its plaque size. Finally, the plaque number and size of the different groups were statistically analyzed using the *t*-test in GraphPad Prism 8.0.2 (San Diego, CA, USA).

For the compound assay, tachyzoites and medium containing TAK-632 (5, 2.5, 1.25, and 0.625 μM), atovaquone (2.5 μM, positive control), or 0.1% DMSO (negative control) were added to each well and cultured for 9 days. The remaining steps are the same as above.

### Invasion assay and intracellular replication assay

For the invasion assay, parasites (1 × 10^6^ tachyzoites per well) were inoculated into HFF cells and infected in 12-well plates for 1 h. Then, tachyzoites without adhesion or invasion were washed with PBS and cultured for 24 h. IFA was performed as described above to observe the number of cells and parasitophorous vacuoles (PVs), which were calculated by randomly selecting five visual fields from the top, bottom, left, right, and center positions. The percentages of invaded parasites were based on the number of PVs divided by the number of HFF cells. For the intracellular replication assay, parasites were cultured and subjected to IFA in the same manner as for the invasion assay; however, the counting method used to assess the intracellular replication assay differed from the invasion assay. Briefly, 100 PVs were randomly counted, and then the number of tachyzoites per PV was determined in the intracellular replication assay.

To examine the effects of compounds on invasion, extracellular tachyzoites (1 × 10^6^) were pretreated with compounds or 0.1% DMSO (negative control) for 3 h at 37 °C and then inoculated into HFF cells for 1 h. The subsequent steps were consistent with the invasion assay described above. To examine the effects of compounds on intracellular replication, at 1 h post-infection (hpi) under standard growth conditions, new medium containing compounds or 0.1% DMSO (negative control) was added to the cells, and then cultured for 24 h. Subsequent steps were consistent with the intracellular replication assay described above. The concentrations of compounds used for the invasion assay and intracellular replication assay were as follows: 5, 2.5, 1.25, and 0.625 μM for TAK-632 and 2.5 μM for atovaquone (positive control). All data were obtained from three independent experiments.

### Egress assay

Parasites were cultured in HFF cells in 12-well plates for 24 h, then treated with 5% absolute ethanol (Zhiyuan Chemical Reagent Co., Ltd., Tianjin, China) for 1 min at room temperature, followed by fixation with 4% paraformaldehyde. IFA was performed with anti-NcSRS2 antibody as described above to assess the egress ability of the parasites. A total of 100 PVs were randomly counted, and the number of ruptured PVs (where tachyzoites egressed from their PVs) was determined: egress ratio = (ruptured PVs/total PVs) × 100% [21, 22]. Three independent trials were carried out.

### Virulence tests in mice

The virulence of the parasites was investigated as described previously [23]. Female BALB/c mice (5 weeks old) were purchased from Beijing Vital River Laboratory Animal Technology Co., Ltd. (Beijing, China, license no. SCXK [Jing] 2021-0006) and housed in ventilated cages under specific-pathogen-free conditions for 7 days prior to the experiment. Five mice per group were injected intraperitoneally with 1.5 × 10^7^ tachyzoites and observed for 30 days. Surviving mice were then humanely euthanized via cervical dislocation, and parasite burden in the brain was determined by quantitative real-time PCR (qPCR) according to the method reported by Collantes-Fernández [24].

### Cytotoxicity assay

The cytotoxicity of compounds was determined on HFFs using the Cell Counting Kit-8 (CCK-8, Dojindo, Beijing, China) according to the manufacturer’s instructions. Briefly, HFF cells were inoculated into 96-well plates at a density of 5000 cells per well and then incubated for 4 h at 37 °C in 5% CO_2_. The compounds were diluted using the twofold serial dilution method. The concentrations of the compounds were as follows: 20, 10, 5, 2.5, 1.25, 0.625, and 0.3125 μM for atovaquone; 100, 50, 25, 12.5, 6.25, 3.125, and 1.5625 μM for pyrimethamine; 198.66, 99.33, 49.665, 24.833, 12.416, 6.2081, 3.1041, and 1.552 μM for penicillin G potassium; 50, 25, 12.5, 6.25, 3.125, 1.5625, and 0.78125 μM for TAK-632. The cells were then exposed to the compounds or 0.1% DMSO (negative control) for 72 h, after which 10 µl CCK-8 reagent was added to each well and reaction was performed for 4 h. The absorbance of the supernatant was measured at 450 nm using a microplate reader (Model 680, Bio-Rad, Richmond, CA, USA). The 50% cytotoxicity concentration (CC_50_) indicates the concentration of compounds that reduced cell growth by 50% and was calculated using the log (inhibitor) versus normalized response-variable slope in GraphPad Prism.

### Parasite division and morphological observation by IFA

HFF cells in 12-well plates were invaded using *N. caninum* Nc1 parasites (5 × 10^5^ tachyzoites per well) for 1 h. Tachyzoites that did not adhere or invade were then washed with PBS. Parasite-infected cells were then treated with 2.5 μM TAK-632 or 0.1% DMSO (negative control) at 37 °C in 5% CO_2_ for 24 h. IFA was performed with mouse anti-inner membrane complex protein 1 (IMC1) (1:100, prepared in our laboratory) and mouse anti-enoyl acyl carrier protein reductase (ENR) antibody (1:100, prepared by Zhang [4]), respectively, to observe the division of the inner membrane complex and apicoplast of tachyzoites [4, 25]. The sequence of TgIMC1 was obtained from ToxoDB (http://toxodb.org/toxo/, accession number: TGGT1_231640) and is listed in Additional file 3: Table S2. Anti-IMC1 was used to label the parental and budding daughter parasites. Rabbit anti-NcSRS2 (1:100, prepared in our laboratory) was used to label parental cells of Nc1 tachyzoites, and Hoechst was used for nuclear staining [26]. A total of 100 PVs were randomly selected to count the number of vacuoles with abnormal divisions: abnormal ratio = (number of abnormal vacuoles/total number of vacuoles) × 100%.

### The in vivo anti-*N. caninum* activity of TAK-632

Freshly egressed tachyzoites of the *N. caninum* Nc1 strain were counted, and then 8-week-old BALB/c mice (six mice per group, half male and half female) were infected by intraperitoneal injection with 1 × 10^7^ parasites. TAK-632 was dissolved in 100% DMSO and diluted in corn oil (Topscience Biotechnology Co., Ltd., Shanghai, China) and then administered to the mice. At 24 hpi, mice were treated with 20, 40, and 60 mg/kg TAK-632 or corn oil (solvent control) once daily for 10 consecutive days. Meanwhile, a blank control group of mice was set up to observe the weight gain of uninfected, untreated mice, and a drug control group of mice treated only with 60 mg/kg TAK-632 was set up to exclude the toxic effects of TAK-632 on mice. Survival and body weight of mice were observed over a period of 30 days. Parasite burden in the brains of surviving mice was determined by qPCR over a 30-day period.

### Statistical analysis

All data were analyzed using GraphPad Prism 8.0.2 (San Diego, CA, USA). IC_50_ and CC_50_ were calculated by the log (inhibitor) versus normalized response-variable slope. Results of intracellular replication assays were analyzed by two-way analysis of variance (ANOVA). Results of survival curves were analyzed by the log-rank (Mantel–Cox) test. Other data were analyzed by *t*-test. Results are expressed as means ± SD, and values of *P* < 0.05 were considered statistically significant.

## Results

### Generation of luciferase-expressing *N. caninum* strain

**Fig. 1:**
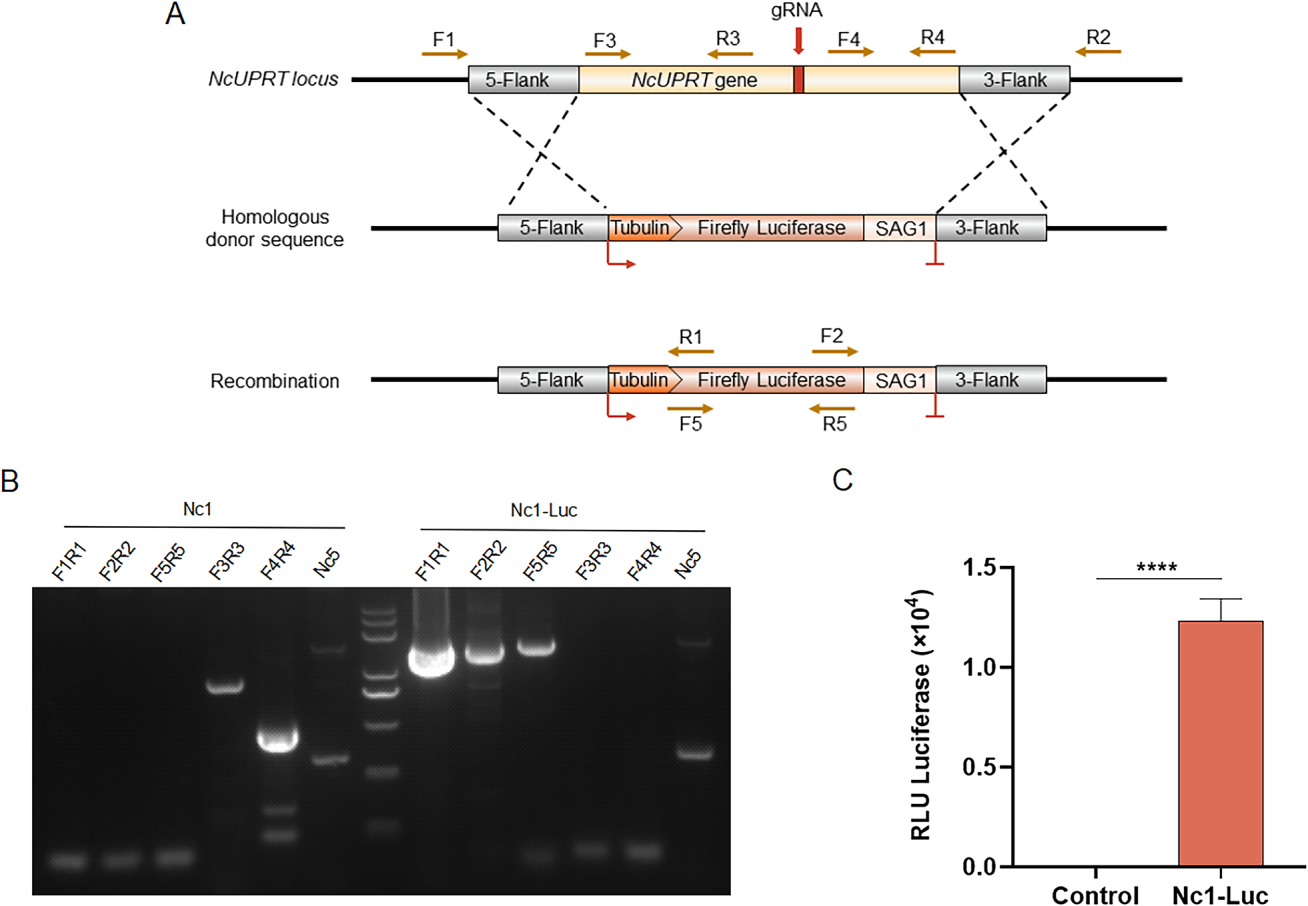
Generation of a luciferase-expressing *N. caninum* strain. **A** Schematic illustration of the replacement of the *NcUPRT* locus with a luciferase expression cassette. The homologous donor sequence contains the luciferase expression cassette driven by the *Tgtubulin* promoter and flanked by regions of homology to *NcUPRT*. **B** Diagnostic PCRs of Nc1-Luc strains. The position of the primers is shown in **A**. Primers F1/R1 and F2/R2 were designed to identify homologous integration based on the products amplified between the luciferase cassette and the external regions of the *NcUPRT* amplicon. The primer F5/R5 was designed to verify the integration of luciferase. Primers F3/R3 and F4/R4 confirm deletion of the *NcUPRT* locus. The *Nc5* gene served as a *N. caninum*-specific gene. **C** Expression of luciferase in Nc1-Luc strains. HFF cells were infected with *N. caninum* tachyzoites (2 × 10^4^), and luciferase activity expressed in relative light units (RLU) was measured at 72 h post-infection. Control: parental Nc1 strain. The assay was performed in triplicate. Statistical analysis was performed by *t*-test in GraphPad Prism (San Diego, CA, USA). ****P < 0.0001

To generate a recombinant *N. caninum* strain expressing firefly luciferase (Nc1-Luc), a luciferase expression cassette was inserted into the *NcUPRT* locus using CRISPR/Cas9 technology, thereby conferring FUDR resistance [19] (Fig. 1A). Briefly, the linearized fragments of pNcUPRT::Luc and plasmid pNc_Cas9CRISPR::sgNcUPRT were co-transfected into the Nc1 strain by electroporation. Parasites were selected using FUDR, and clones were screened by the limiting dilution method. Diagnostic PCRs were used to identify the correct integration of luciferase at the *NcUPRT* locus in the clones (Fig. 1B). Next, luciferase activity assay was performed to verify the expression of firefly luciferase in Nc1-Luc stains. The results showed that the Nc1-Luc strains had a high luminescence signal, whereas the Nc1 strains did not, indicating the expression of firefly luciferase in the Nc1-Luc strains (Fig. 1C).

### The growth properties of Nc1-Luc in vitro

The growth phenotype of the constructed strains could not be altered because there was a need to construct a base strain that could be used for drug screening. *Neospora caninum* tachyzoites undergo a complete lytic cycle in host cells, including invasion into cells, intracellular proliferation, and egress from cells [27]. Plaque formation can comprehensively reflect the growth of *N. caninum* tachyzoites throughout the lytic cycle [19]. Therefore, to evaluate whether luciferase expression affects the growth of *N. caninum* in vitro, plaque formation, invasion, intracellular proliferation, and egress assays were performed.

**Fig. 2:**
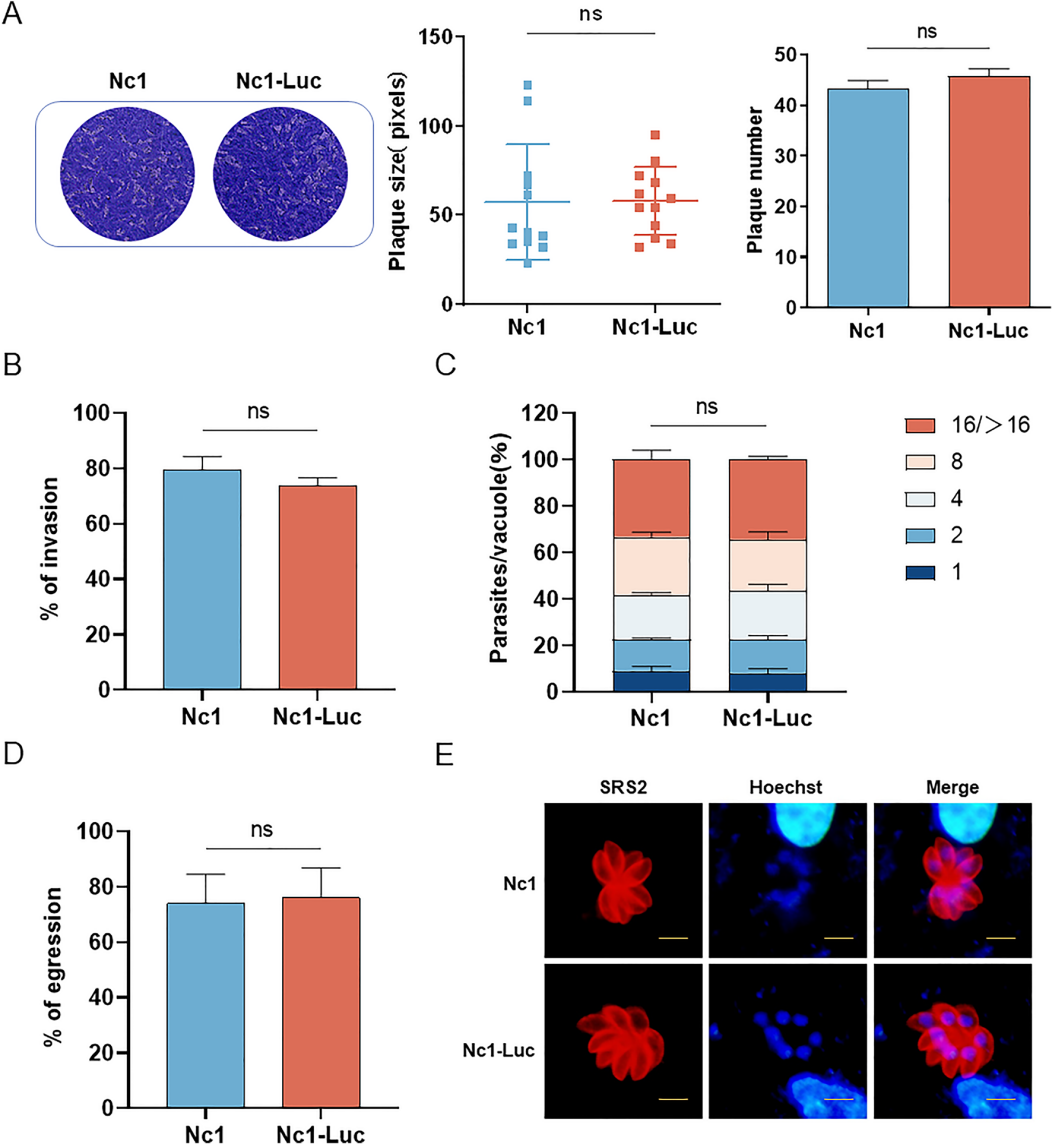
The non-effect of luciferase expression on the growth of *N. caninum* in vitro. **A** Plaque formation assay; 300 tachyzoites were inoculated into HFF cells, cultured for 9 days, then fixed with 4% paraformaldehyde and stained with 0.2% crystal violet. The total number of plaques in each well was manually counted, and at least 10 plaques per well were randomly selected to measure the pixel size of each plaque using pixels in Photoshop CC software (Adobe, USA) to represent its plaque size. Three independent experiments were performed, and the plaque number represented the average number of plaques for the three wells. **B** Invasion assay. The numbers of PVs and HFF cells were calculated by randomly selecting five visual fields. The percentages of invaded parasites were based on the number of PVs divided by the number of HFF cells in one field. **C** Intracellular proliferation assay. A total of 100 PVs were randomly selected, and the number of tachyzoites per PV was counted. **D** Egress assay. The egress ability of the parasites was assessed after treatment with 5% absolute ethanol. A total of 100 PVs were randomly counted, and the number of ruptured PVs was determined. Egress ratio = (ruptured PVs/total PVs) × 100%. **E** Morphological characteristics of Nc1-Luc and Nc1 detected by IFA. Shapes of parasites were visualized using anti-NcSRS2 (red), and nuclear DNA was stained using Hoechst (blue). Scale bar = 5 μm. The results of plaque formation, invasion, and egress assays were analyzed by *t*-test. The intracellular proliferation assay was analyzed by two-way ANOVA in GraphPad Prism (San Diego, CA, USA). ns: no difference, *P* > 0.05

The plaque formation assay showed that Nc1-Luc strains were capable of forming plaques normally, and the sizes and numbers of plaques were not significantly different from those of the Nc1 strains (Fig. 2A). The invasion assay showed that both Nc1-Luc and Nc1 strains were able to invade host cells without barriers (Fig. 2B). The intracellular proliferation assay showed that Nc1-Luc strains could form PVs within cells and proliferate in numbers of 2, 4, 8, 16, or more, as did Nc1 strains (Fig. 2C). For the egress assay, Nc1 and Nc1-Luc were simultaneously stimulated with 5% absolute ethanol. The results showed that Nc1-Luc was able to escape from the cells at a speed similar to that of Nc1. (Fig. 2D). In addition, the morphological characteristics of Nc1-Luc were observed by fluorescence microscopy. The results showed that Nc1-Luc strains undergo normal binary fission, with tachyzoites having a classical crescent shape (Fig. 2E). Overall, these results indicated that luciferase expression has no effect on the in vitro growth of *N. caninum*.

### The pathogenicity of Nc1-Luc in mice

**Fig. 3:**
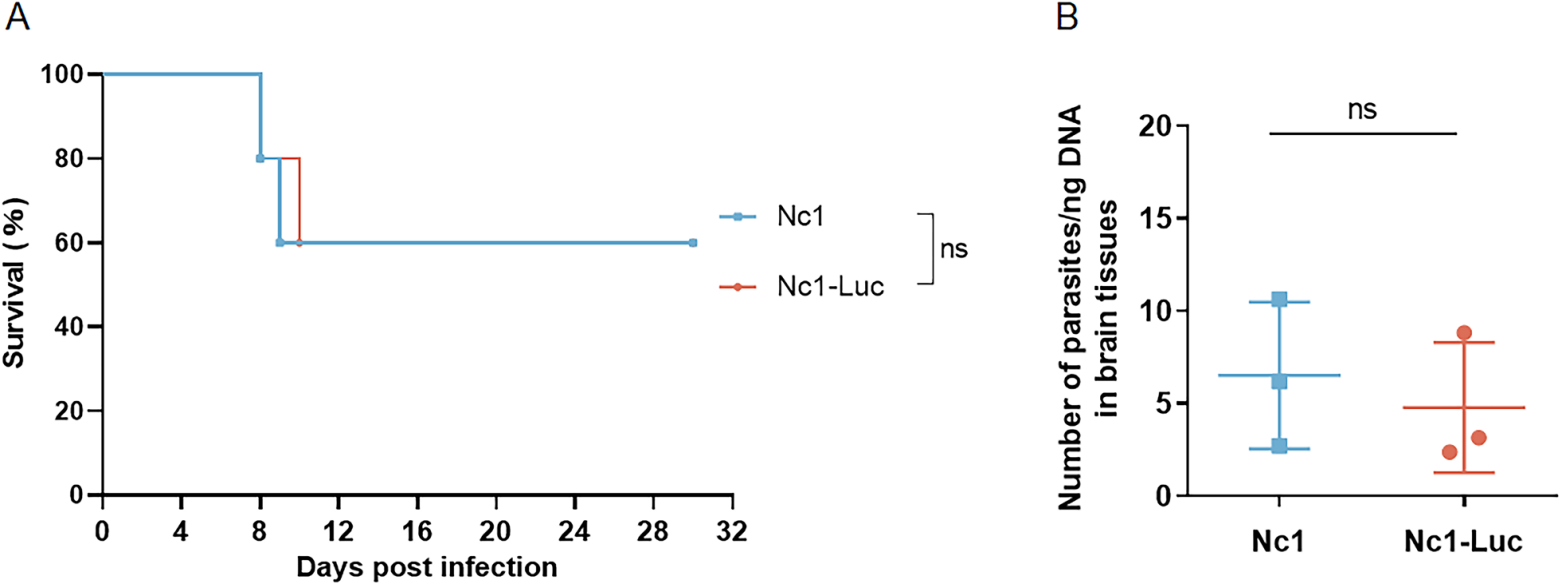
The non-effects of luciferase expression on the pathogenicity of *N. caninum* in mice. **A** Survival curves of mice. 1.5 × 10^7^ tachyzoites were inoculated intraperitoneally into BALB/c mice (5 mice per group), and the survival of mice was observed over a 30-day period. **B** Parasite burden in the brain. After 30 days of infection, the parasite burden of surviving mice was detected by qPCR. The qPCR assay was performed in triplicate. Results are shown as mean ± SD. Survival curves were analyzed using the log-rank (Mantel–Cox) test, and qPCR results were analyzed by *t*-test in GraphPad Prism (San Diego, CA, USA). ns: no difference, *P* > 0.05

To evaluate whether luciferase expression affects the pathogenicity of *N. caninum* in mice, 1.5 × 10^7^ Nc1-Luc and Nc1 tachyzoites were inoculated intraperitoneally into BALB/c mice, and the mice were observed for 30 days. Both groups of mice showed obvious clinical symptoms of depression and bowed back on the fourth day. In the Nc1-Luc group, two out of five mice died at 8 days post-infection (dpi) and 10 dpi, respectively. In the Nc1 group, two mice died at 8 dpi and 9 dpi, respectively. The survival rate of tachyzoite infection in both groups was 60%; thus, there was no significant difference between the two groups (Fig. 3A). After 30 days, surviving mice were humanely euthanized via cervical dislocation. Parasite burden in the brain was then detected using qPCR, which showed no significant difference between the two groups (Fig. 3B). This suggests that luciferase expression does not affect the pathogenicity of *N. caninum* in mice.

### Determination of the sensitivity of Nc1-Luc to known drugs

**Fig. 4:**
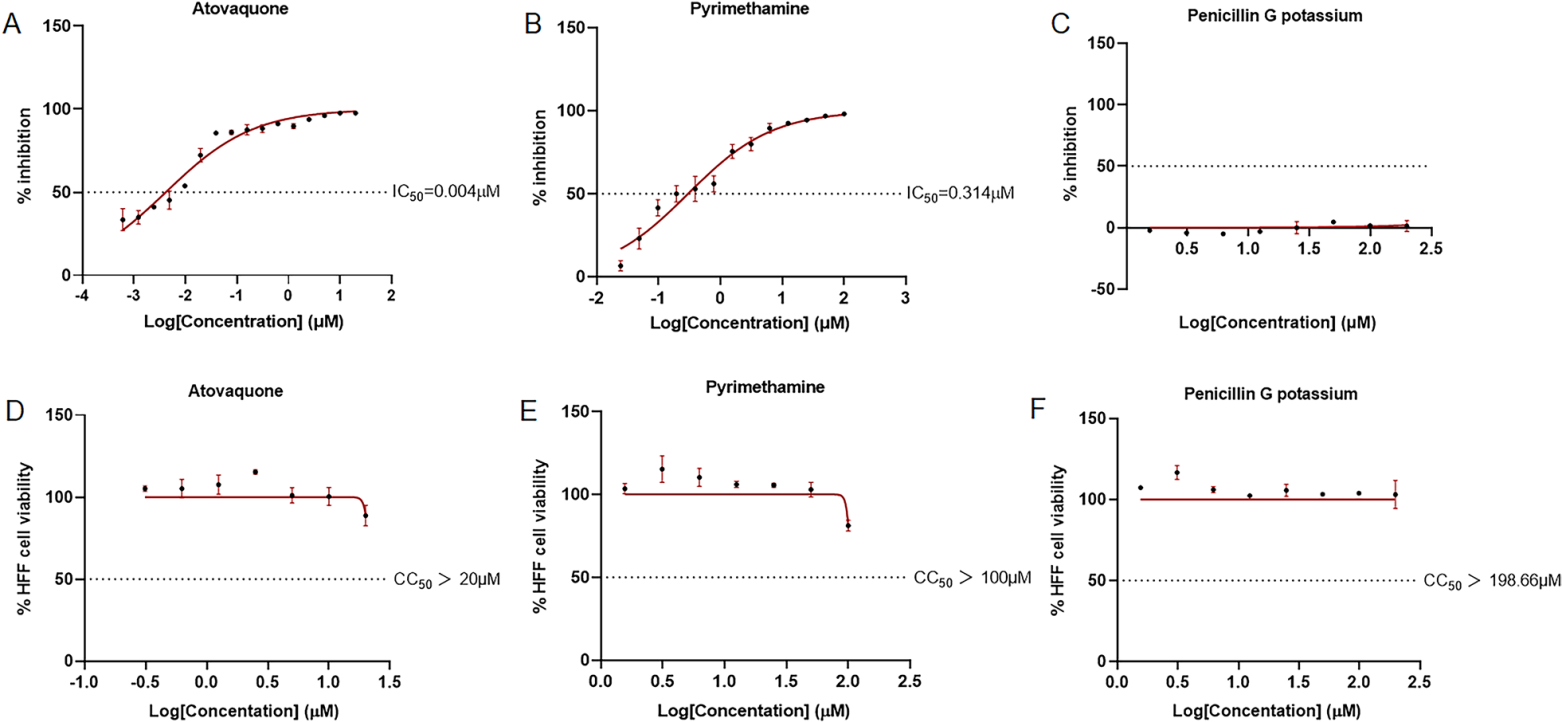
In vitro anti-*N. caninum* determination using Nc1-Luc as a tool. **A**–**C** Inhibition of *N. caninum* (Nc1-Luc) after treatment with atovaquone (**A**), pyrimethamine (**B**), or penicillin G potassium (**C**). Nc1-Luc strains were treated with different concentrations of drugs, and RLUs were detected after 72 h. **D**–**F** Cell viability of HFF cells upon treatment with atovaquone (**D**), pyrimethamine (**E**), or penicillin G potassium (**F**). HFF cells were treated with different concentrations of drugs, and absorbance was measured at 450 nm after 72 h. Results are shown as mean ± SD from three independent assays. IC_50_ and CC_50_ were calculated using the log (inhibitor) versus normalized response-variable slope in GraphPad Prism (San Diego, CA, USA)

The ideal base strain for drug screening should be drug-sensitive. Accordingly, the next focus was on testing the Nc1-Luc strain as a tool for evaluating the efficacy of anti-*N. caninum* therapeutics. Since atovaquone and pyrimethamine have been shown to effectively inhibit the growth of *N. caninum* [28, 29], and penicillin is ineffective against protozoa [30], atovaquone and pyrimethamine were chosen as positive drugs and penicillin G potassium as a negative drug to test their inhibitory effects on *N. caninum* using the Nc1-Luc strain. Briefly, HFF cells were infected with Nc1-Luc and then cultured in the absence or presence of serial dilutions of atovaquone, pyrimethamine, or penicillin G potassium, respectively. After 72 h of treatment, the luciferase activity of Nc1-Luc was detected. The results showed that both atovaquone and pyrimidine inhibited the growth of *N. caninum* in a dose-dependent manner (Fig. 4A, B). In contrast, penicillin G potassium did not inhibit the growth of *N. caninum* even at the highest concentration (Fig. 4C). Consistent with previously published studies [28, 29], the IC_50_ values were 0.004 ± 0.0007 µM for atovaquone and 0.314 ± 0.1040 µM for pyrimidine. To assess the cytotoxicity of these drugs on HFF cells, dilutions of the drugs were sequentially added to the HFF cells, and cell viability was determined using CCK-8 reagent. The results showed that no obvious cytotoxicity was detected for any of the three drugs (Fig. 4D, E, F). Taken together, our results indicate that Nc1-Luc strains maintain sensitivity to drugs and can be used as an effective tool for in vitro drug screening of *N. caninum*.

### Evaluation of anti-*N. caninum* activity of TAK-632 in vitro

**Fig. 5:**
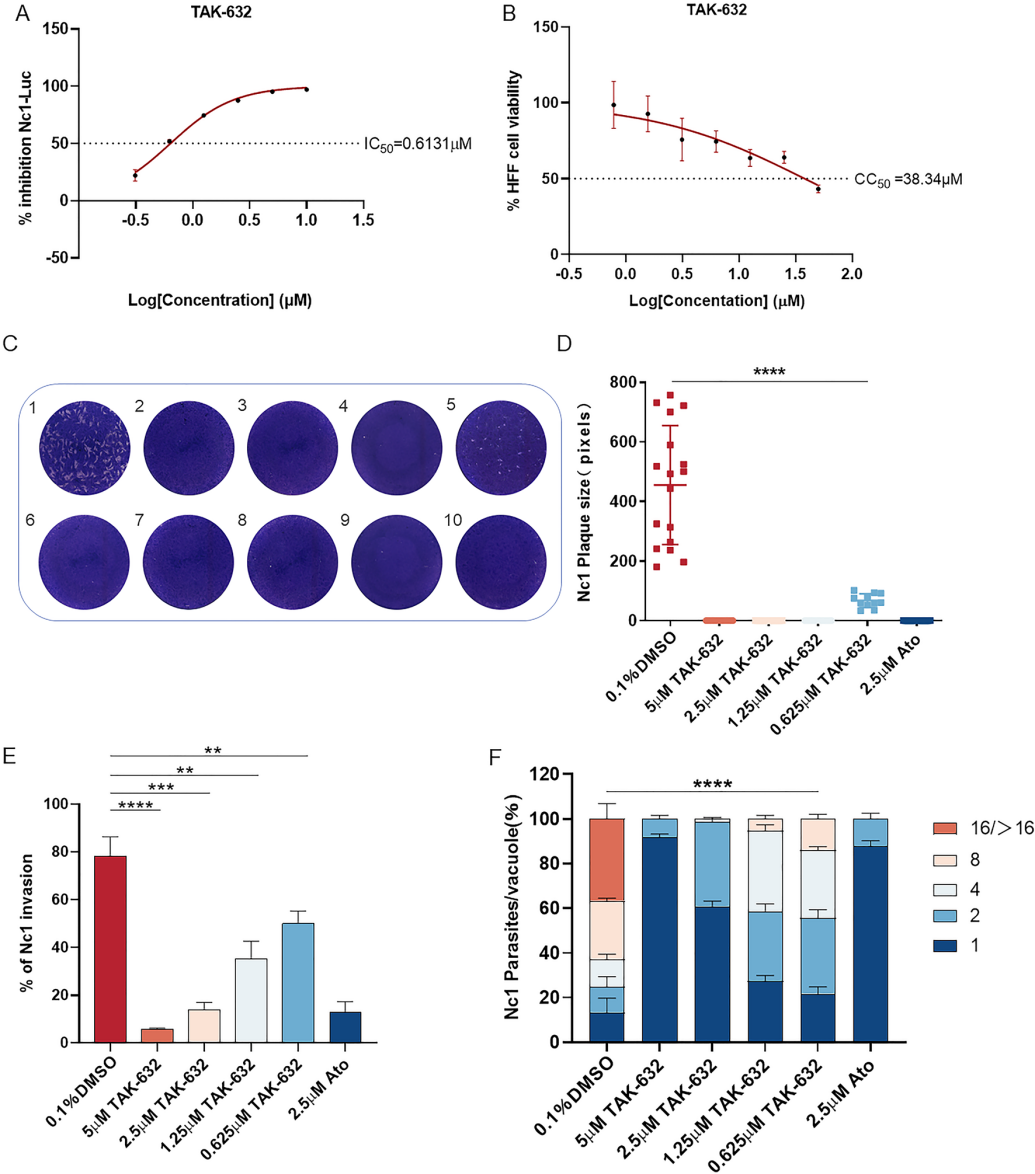
Dose-dependent inhibition of *N. caninum* tachyzoites displayed by TAK-632 in vitro. **A** The inhibition curve of TAK-632 against *N. caninum*. The IC_50_ was calculated using the log (inhibitor) versus normalized response-variable slope in GraphPad Prism (San Diego, CA, USA). **B** HFF cell viability upon treatment with TAK-632. The CC_50_ for TAK-632 was calculated as 38.34 μM. **C**, **D** Plaque formation assay. 300 tachyzoites were inoculated into HFF cells. The infected cells were treated with different drugs. Cells were cultured for 9 days, and then fixed with 4% paraformaldehyde and stained with 0.2% crystal violet. At least 10 plaques per well were randomly selected to measure the pixel size of each plaque using pixels in Photoshop CC software (Adobe, USA) to represent its plaque size. 1:0.1% DMSO; 2–5: 5, 2.5, 1.25, and 0.625 μM TAK-632; 6: 2.5 μM atovaquone; 7–10: HFF cells were not infected with tachyzoites and treated with 5, 2.5, 1.25 and 0.625 μM TAK-632, respectively. **E** Invasion assay. Extracellular tachyzoites were pretreated with different drugs for 3 h at 37 °C, followed by invasion for 1 h, and IFA was performed to determine the percentage of invaded parasites. **F** Intracellular proliferation assay. After infecting HFF cells with tachyzoites for 1 h, the infected cells were treated with different drugs. The proportion of vacuoles containing 2, 4, 8, and 16 tachyzoites was calculated by IFA. Results are shown as mean ± SD. Each assay was performed in triplicate. The results of plaque formation and invasion assays were analyzed by *t*-test. Intracellular proliferation assays were analyzed by two-way ANOVA in GraphPad Prism (San Diego, CA, USA). ****P < 0.0001; ***P < 0.001; **P < 0.01

TAK-632, a pan-RAF inhibitor, has been reported to be active against melanoma and necroptosis [15–18]. However, it remains unclear whether TAK-632 possesses anti-*N. caninum* activity. Therefore, the Nc1-Luc strain was used to determine the effect of different concentrations of TAK-632 on *N. caninum* tachyzoites. HFF cells were treated with different concentrations of TAK-632 after infection with the Nc1-Luc strain. Luciferase activity was measured at 72 hpi. The results showed that TAK-632 was able to inhibit the growth of *N. caninum* in a dose-dependent manner (Fig. 5A), with an IC_50_ value of 0.6131 ± 0.03743 µM, whereas no significant toxicity was observed in the therapeutic concentration range (Fig. 5B). The CC_50_ value of TAK-632 on HFF cells was 38.34 ± 1.465 µM. The selectivity index (SI, CC_50_/IC_50_) of a compound expresses the efficacy of a compound to selectively inhibit a pathogen in vitro [31]. The SI of TAK-632 was 62.53, indicating a higher selectivity of TAK-632 against *N. caninum* than host cells.

The plaque formation assay was used to comprehensively evaluate the growth of *N. caninum* tachyzoites at different concentrations of TAK-632 treatment during the entire lytic cycle (DMSO, negative control; atovaquone, positive control). The results showed no plaque formation after 5, 2.5, and 1.25 μM TAK-632 treatments, which was significantly different from the DMSO control group (Fig. 5C). When treated with 0.625 μM TAK-632, visible plaques were formed, but the plaque size was significantly smaller than that of the DMSO control group (Fig. 5D). In addition, 5, 2.5, 1.25, and 0.625 μM TAK-632 were added to HFF cells uninfected with tachyzoites, demonstrating an absence of TAK-632 affecting cell growth (Fig. 5C). The reduction in plaque formation could be caused by impairment of one or more steps of the lytic cycle. Therefore, an investigation was performed next to determine which lytic cycle of *N. caninum* was affected by TAK-632. Parasite invasion and intracellular proliferation processes were assessed. For parasite invasion, tachyzoites were pretreated with different concentrations of TAK-632 for 3 h, after which the drug was removed and tachyzoites invaded the cells for 1 h. The invasion rate of tachyzoites in the DMSO control group was 77%. After treatment with TAK-632, the invasion rate of tachyzoites decreased significantly with increasing drug concentration, and the invasion rates of tachyzoites after treatment with 5, 2.5, 1.25, and 0.625 μM TAK-632 were 13%, 14%, 35%, and 50%, respectively (Fig. 5E). For parasite intracellular proliferation, different concentrations of TAK-632 were added to the cell wells at 1 h of tachyzoite invasion, followed by 24 h of culture. The results showed that in the DMSO control group, the number of tachyzoites in the PV was 2, 4, 8 and 16, respectively, with 8 and 16 tachyzoites dominating. With an increase in TAK-632 concentration, the number of tachyzoites gradually decreased and proliferation disorders occurred, which was significantly different from the control group. After treatment with 5 and 2.5 μM TAK-632, tachyzoites were mostly single in PV. After treatment with 1.25 and 0.625 μM TAK-632, most of the PVs contained two or four tachyzoites. The results showed that TAK-632 potently inhibited the intracellular proliferation of *N. caninum* (Fig. 5F). Taken together, our results indicate that TAK-632 effectively inhibits the growth of *N. caninum* in vitro.

### Treatment with TAK-632 causes abnormal morphology and division of *N. caninum*

**Fig. 6:**
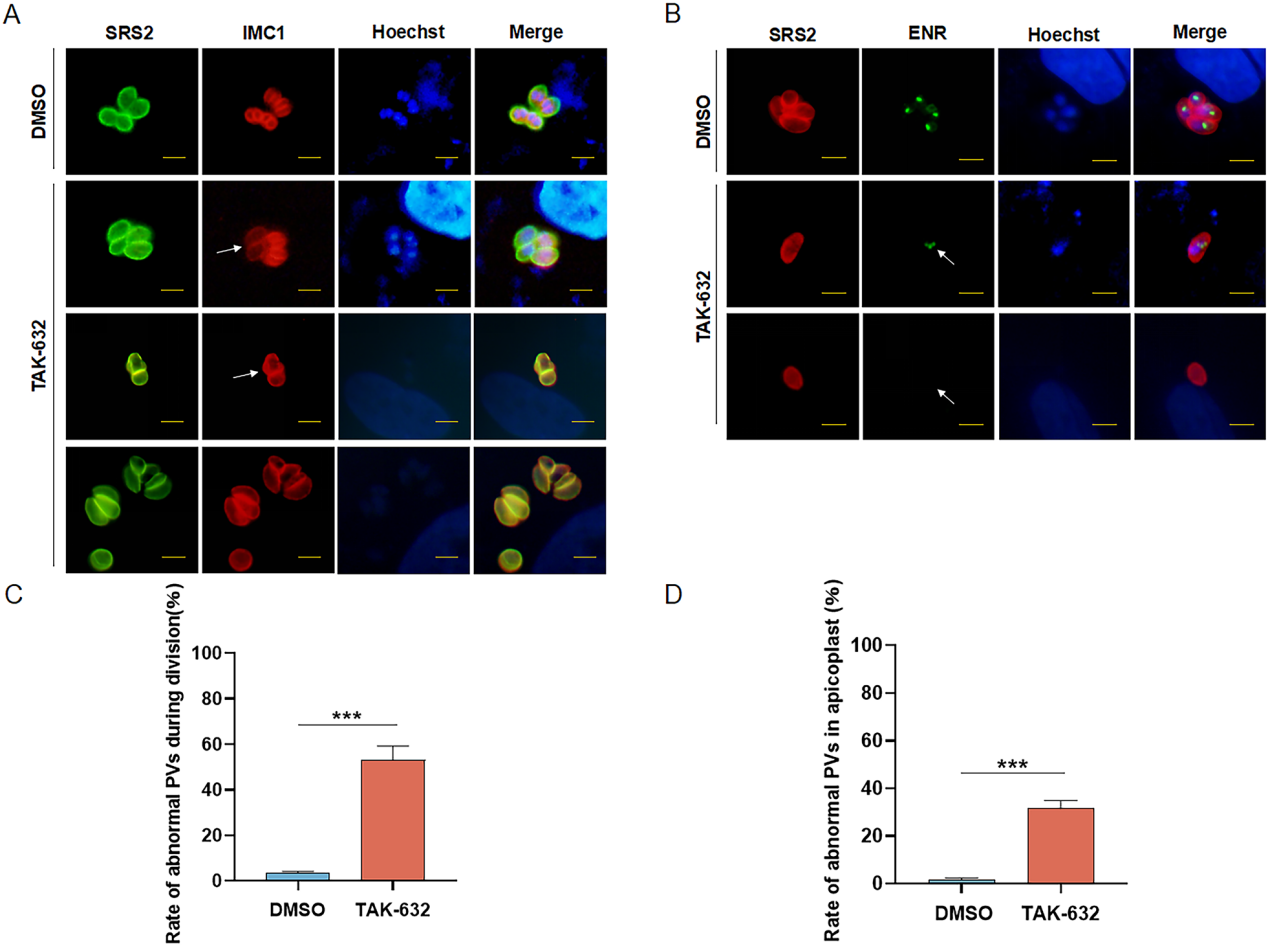
Effects of TAK-632 on the morphology and division of *N. caninum*. HFFs were invaded using 5 × 10^5^ Nc1 tachyzoites and then treated with DMSO or TAK-632. **A** IFA observation of IMC division of tachyzoites by. SRS2 was used to label parental cells of Nc1 tachyzoites. IMC1 was used to label both parental and budding daughter cells. Hoechst was used to stain the nuclei. Arrows indicate tachyzoites with abnormal IMC division. Scale bar, 5 μm. **B** IFA observation of apicoplast division of tachyzoites. ENR was used to label the apicoplasts of Nc1 tachyzoites. Arrows indicate tachyzoites with abnormal apicoplasts. Scale bar, 5 μm. **C** Proportion of vacuoles with abnormal IMC divisions. The field of view was randomly selected to count the number of vacuoles with abnormal IMC division. Abnormal ratio = (number of abnormal vacuoles/total number of vacuoles) × 100%. **D** Proportion of vacuoles with abnormal apicoplast division. Results were tested by *t*-test. ***P < 0.001

Considering the inhibitory effect of TAK-632 on the growth of *N. caninum*, we evaluated whether treatment with TAK-632 would alter the morphology and division of *N. caninum* tachyzoites. IMC1 and ENR are often used to mark the division of *N. caninum*, because their division is often accompanied by the budding of daughter parasites [4, 25]. The inner membrane complex and apicoplast of *N. caninum* tachyzoites were visualized by IFA using anti-IMC1 and anti-ENR antibodies, respectively. In the DMSO group, the morphology and division of the tachyzoites were normal. Different types of unnatural divisions and morphology were observed after 24 h of treatment with TAK-632. For the inner membrane complex, IMC1 division of tachyzoites in the same PV was asynchronous (Fig. 6A). Tachyzoite morphology was also affected by both rounded and swollen shapes (Fig. 6A). In addition, odd numbers of tachyzoites (e.g., one, three, or five tachyzoites) were found to exist in a single PV (Fig. 6A). For the apicoplast, more than two apicoplasts were observed during some tachyzoite divisions, while others had no apicoplasts (Fig. 6B). Statistically, 53% of PVs were found to have abnormal inner membrane complexes, and 31% of PVs were found to have abnormal apicoplasts (Fig. 6 C, D). These results suggest that TAK-632 affects the morphology and division of *N. caninum*, which may be related to its mechanism of anti-*N. caninum* activity.

### Evaluation of anti-*N. caninum* activity of TAK-632 in mice

**Fig. 7:**
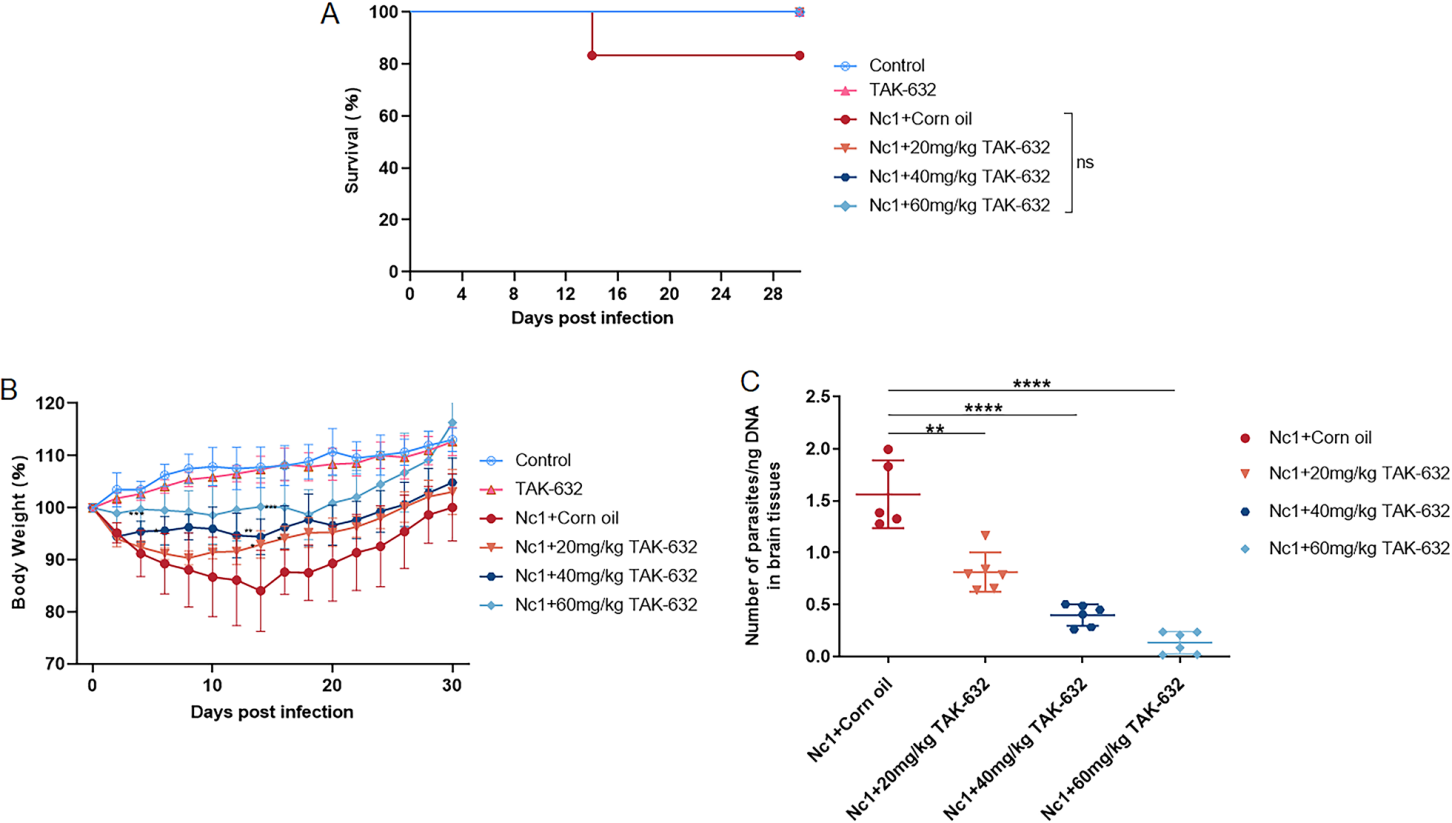
Therapeutic effect of TAK-632 on *N. caninum* infection in mice. BALB/c mice were infected with a dose of 1 × 10^7^ Nc1 tachyzoites by intraperitoneal injection. After 24 h of infection, mice were orally administered corn oil or TAK-632 once daily for 10 consecutive days at the indicated concentrations (six mice per group). Mice were observed for 30 days. **A** Survival curves. **B** Body weight (%). The weight was measured every 2 days. **C** Detection of parasite burden in the brains of surviving mice by qPCR. Results are shown as mean ± SD. Survival curves were plotted with GraphPad Prism using the log-rank (Mantel–Cox) test (San Diego, CA). Body weight and parasite burden in the brain were analyzed by *t*-test in GraphPad Prism (San Diego, CA, USA). **** P<  0.0001; ***P < 0.001; **P < 0.01; *P < 0.05

To evaluate the effects of TAK-632 on *N. caninum* in vivo, four experimental groups of BALB/c mice (6 mice/group) were infected intraperitoneally with 1 × 10^7^ Nc1 tachyzoites per mouse and orally administered either corn oil (solvent control) or TAK-632 at the indicated concentrations once a day for 10 consecutive days. The blank control group was set up to observe the weight gain of the uninfected, untreated mice. The TAK-632 group was treated with only 60 mg/kg TAK-632 to exclude the toxic effect of TAK-632 on mice. Treatment with TAK-632 increased the survival rate during *N. caninum* infection, but the difference was not significant compared with the corn oil group (Fig. 7A). Infected mice exhibited a lower rate of weight loss after treatment with TAK-632 (Fig. 7B). Unsurprisingly, the group treated only with TAK-632 gained weight and were in good physical condition, which was consistent with that of the blank control group. In addition, parasite burden in the brains of surviving mice was determined by qPCR over a 30-day period. TAK-632 treatment significantly reduced parasite burden in the brain relative to the corn oil group (Fig. 7C). Compared with the Nc1+corn oil group, TAK-632 treatment at 20, 40, and 60 mg/kg reduced parasite burden by fivefold, 12-fold, and 38-fold, respectively. These results suggest that TAK-632 treatment controls *N. caninum* infection in mice.

## Discussion

Neosporosis is a major cause of abortion in cattle worldwide, and there is an urgent need to develop new therapeutic approaches to control neosporosis [1–3]. Traditional drug screening methods for determining the IC_50_ against *N. caninum* have been described in the literature [4–7]. However, most of these methods are cumbersome, time-consuming, and labor-intensive. Therefore, it is important to develop efficient, rapid, and high-throughput drug evaluation and screening methods for *N. caninum.* Reporter genes have facilitated drug discovery in many protozoan studies. As an excellent reporter gene, luciferase has been widely used in drug screening due to its advantages of high sensitivity, strong specificity, and fast detection [11]. In this study, a luciferase-expressing *N. caninum* strain was constructed to evaluate the infectivity of the strain and its feasibility in anti-*N. caninum* drug screening.

We chose to use the CRISPR/Cas9 technology platform to insert luciferase at the *NcUPRT* locus because its inactivation leads to FUDR resistance, thus providing a convenient assay for measuring gene disruption without affecting parasite viability [32]. The luciferase activity assay showed high luminescence signals of Nc1-Luc strains, demonstrating the high expression of luciferase in the parasites. Importantly, the growth properties of the parental strains Nc1 and Nc1-Luc were compared in vitro, and similar lytic cycles and morphological characteristics were observed. Virulence tests in mice showed that the pathogenicity of the Nc1-Luc strain was unaltered. These results indicate that Nc1-Luc strains do not lose virulence during luciferase expression and can replace Nc1 in causing *N. caninum* infections. In addition, Nc1-Luc strains maintained a high luminescence signal after serial passages in vitro or recovery from infected BALB/c mice, demonstrating that luciferase expression was stable in Nc1-Luc strains (results not shown).

The Nc1-Luc strains were then treated with the known anti-*N. caninum* drugs atovaquone and pyrimethamine. The results showed that atovaquone and pyrimethamine exerted a dose-dependent inhibitory effect on Nc1-Luc, which was reflected by a reduced level of luminescent signaling. Pereira et al. [28, 29] demonstrated that atovaquone and pyrimethamine inhibited the growth of *N. caninum*, with IC_50_ values of 0.008 ± 0.002 μM and 0.312 ± 0.017 μM, respectively. This is consistent with our findings that atovaquone and pyrimethamine inhibited Nc1-Luc strains, with IC_50_ values of 0.004 ± 0.0007 µM and 0.314 ± 0.1040 µM, respectively, demonstrating high susceptibility of Nc1-Luc strains to drug evaluation. Moreover, Pereira et al. [28, 29] used *N. caninum* expressing β-gal (Nc1-LacZ), which required incubation of the lysates with the substrate for 4 h, whereas our Nc1-Luc strain could be detected immediately after mixing of the lysates with the substrate. Therefore, this method using luciferase reduces the time required for in vitro screening of compounds against *N. caninum*. Bioluminescence imaging can correlate specific cell activity with in vivo biological luminescence signals to track cell dynamic changes in real time, often using a luciferase reporting system [33]. The use of recombinant parasites expressing luciferase for bioluminescence imaging allows visualization of the parasite infection process in animals and also provides stronger evidence for drug screening [34]. Lourido et al. [35] monitored the process of *Toxoplasma* infection by bioluminescence imaging using the ME49 strain expressing firefly luciferase, and found that compound 24 limited the transmission of *Toxoplasma* in mice, reduced parasite burden in the brain, and prevented reactivation of chronic toxoplasmosis. However, our lack of an in vivo imaging platform limits the current study. As a next step, the feasibility of Nc1-Luc for in vivo drug screening will be explored if the conditions are available.

Using the Nc1-Luc strains, a mammalian RAF kinase inhibitor, TAK-632, was shown to be active against *N. caninum*, with an IC_50_ of 0.6131 ± 0.03743 μM. Cytotoxicity analyses of TAK-632 showed that effective concentrations of TAK-632 against the parasite were innocuous to the host cells, as the CC_50_ obtained for TAK-632 (38.34 ± 1.465 µM) was 62.53-fold higher than the IC_50_ obtained for *N. caninum*. The study subsequently showed that TAK-632 inhibited the growth of *N. caninum* by impairing the lytic cycle, mainly by inactivation of extracellular invasion and slowing of intracellular replication, resulting in unformed plaques. Plaque assays showed no damage or shedding in cells treated directly with TAK-632, suggesting that TAK-632 acts directly on *N. caninum* to inhibit the formation of plaque rather than indirectly on infected cells, and the results of the invasion assays confirmed this. Importantly, TAK-632 also inhibited *N. caninum* in vivo, indicating a slowing of weight loss and a reduction in parasite burden in mice. However, we were unable to determine whether TAK-632 acts on parasites by crossing the blood–brain barrier or whether it first kills the parasites and then reduces the number of parasites entering the brain tissue. In many studies, recombinant parasites expressing luciferase have been utilized to monitor the number and infection site of parasites in mice after drug treatment in real time, which provides a reliable method to address our questions for further study [11, 34–39].

The inclusion of a positive drug control in in vivo experiments plays an important role in providing a comparative baseline for evaluating the effectiveness of the tested compound and bolstering credibility by excluding false positives [40–43]. Thus, the lack of a positive drug control would impose some limitations regarding in vivo experiments. It should be noted that pyrimethamine treatment was additionally performed on *N. caninum*-infected mice, and although the parasite burden was not assessed, the survival rate was comparable to that obtained with TAK-632 treatment (data not shown). In the next step, we will improve the in vivo experiments by incorporating the positive drug control into the in vivo experiment of TAK-632 to compare the therapeutic effect of the positive drug and TAK-632 under the same experimental conditions to obtain more meaningful results.

On the other hand, *N. caninum* are single-celled eukaryotes with tachyzoites in multiples of 2, and tachyzoites in the same PV usually divide synchronously [44]. However, *N. caninum* tachyzoites treated with TAK-632 exhibited a large number of abnormal divisions, such as asynchronous divisions, and even three or five tachyzoites in one PV. This may be one of the reasons why TAK-632 affects the proliferation of *N. caninum*. The slower proliferation of *N. caninum* tachyzoites may be related to their conversion to bradyzoites, which can be induced by endogenous or exogenous factors, including drug effects. Studies have found that 60 μg/ml tylosin in vitro induces about 10–15% cyst formation of *N. caninum* tachyzoites [45]. Tissue cysts containing bradyzoites can resist attack by the host immune system and drugs, allowing the parasite to exist in the host for a long period and cause chronic infection [45]. Therefore, the effect of TAK-632 on bradyzoite transformation and proliferation of *N. caninum* bradyzoites in vitro is of great importance and should be further investigated. The anti-melanoma target of TAK-632 is the B-RAF kinase in the mitogen-activated protein kinase (MAPK) pathway [15, 16]. Unlike mammalian cells, the MAPK pathway in apicomplexan parasites has not been thoroughly analyzed [46]. Attempts were made to find B-RAF homologs in *N. caninum*, but to no avail. In addition, TAK-632 inhibits other kinases, such as RIPK1 and RIPK3 [17, 18]. This suggests that TAK-632 may target other kinases for anti-*N. caninum* effects. The apicoplast is involved in the synthesis of nutrients necessary for *N. caninum*; it plays an extremely important role in the viability of parasites, and is absent in mammalian cells. Thus, it is regarded as an ideal target for drug development in many apicomplexan protozoa [4, 47]. This study found that TAK-632 causes abnormal division or absence of the apicoplast in *N. caninum*, which provides insight for the identification of anti-*N. caninum* targets of TAK-632. Transcriptomic, proteomic, and metabolomic studies should be performed on treated parasites to determine future steps to be taken to achieve a better understanding of the antiparasitic mechanism.

Promising therapeutic results with TAK-632 in mouse models indicate the potential value of this chemotherapeutic approach for neosporosis in other animals, such as cattle. In the case of neosporosis in cattle, the ideal therapeutic options should be highly effective and economical. However, the high price and lack of therapeutic efficacy of TAK-632 compared with existing drugs limit its potential as a market drug. Zhang et al. [18] performed structural optimization of TAK-632 to enhance its anti-necroptosis activity. Okaniwa et al. [15] prepared a solid dispersion formulation of TAK-632, which demonstrated high bioavailability in rats and dogs. Therefore, the in vivo therapeutic effect of TAK-632 can be improved in future studies by optimizing the molecular structure and modifying the formulation. In addition, co-application of TAK-632 in combination with other drugs or vaccines may be considered to enhance the effect and reduce the cost [48].

## Conclusions

The luciferase-expressing strain of *N. caninum* can be used as an effective tool for drug screening and related studies of *N. caninum*. Furthermore, the selective pan-RAF inhibitor TAK-632 inhibits the growth of *N. caninum* and is considered a leading candidate compound for the new treatment of neosporosis.

### Supplementary Information

**Additional file 1: Table S1.** Primers used for this study.

**Additional file 2: Figure S1.** Construction of pEASY-Luc and pNcUPRT::Luc plasmids. A. Diagram of the pEASY-mCherry plasmid structure. The pEASY-mCherry plasmid contained the CAT-mCherry expression cassette driven by the *Tgtubulin* promoter and terminated by the *TgSAG1* terminator*.* This plasmid was constructed and maintained in our laboratory. B. PCR products used to construct the pEASY-Luc plasmid. The firefly luciferase fragment was amplified from commercial plasmid pGL3-Basic Vectors. The vector fragment with the *Tgtubulin* promoter and the *TgSAG1* terminator (Tubulin-SAG1) was amplified from the pEASY-mCherry plasmid. C. Diagram of the pEASY-Luc plasmid structure which was based on the pEASY-mCherry plasmid by replacing the firefly luciferase at the CAT-mcherry position. D. PCR products used to construct the pNcUPRT::Luc plasmid. The pUC19 vector fragment with the upstream and downstream homology arms of the *NcUPRT* gene was amplified from the pNcUPRT::GFP plasmid constructed by Yang [19]. The firefly luciferase expression cassette (T-Luc-S) was amplified from the previously constructed pEASY-Luc plasmid. E. Diagram of the pNcUPRT::Luc plasmid structure which was based on the pNcUPRT::GFP plasmid by replacing the firefly luciferase expression cassette at the position of the GFP expression cassette.

**Additional file 3: Table S2.** The information on NcSRS2, TgIMC1, *TgTubulin* promoter, and *TgSAG1* terminator.

## Data Availability

All datasets generated for this study are included in the manuscript and its additional files.

## Abbreviations

*N. caninum*: *Neospora caninum*
*T. gondii*: *Toxoplasma gondii*
CRISPR/Cas9: Clustered regularly interspaced short palindromic repeats (CRISPR)-associated protein 9
Pan-RAF: Pan-rapidly accelerated fibrosarcoma
UPRT: Uracil phosphoribosyltransferase
IC_50_: Half-maximal inhibitory concentration
CC_50_: 50% Cytotoxicity concentration
SI: Selectivity index
Luc: Luciferase
GFP: Green fluorescent protein
β-gal: β-Galactosidase
DMSO: Dimethyl sulfoxide
FUDR: Fluorodeoxyribose
RLU: Relative light unit
PVs: Parasitophorous vacuoles
IMC1: Inner membrane complex protein 1
ENR: Enoyl acyl carrier protein reductase
IFA: Immunofluorescence assay
qPCR: Quantitative real-time polymerase chain reaction
Vero: African green monkey kidney cells
HFF: Human foreskin fibroblasts
DMEM: Dulbecco’s modified Eagle medium
FBS: Fetal bovine serum
BSA: Bovine serum albumin
PBS: Phosphate-buffered saline
